# The Formation of the Strength of Castings including Stress and Strain Analysis

**DOI:** 10.3390/ma17112484

**Published:** 2024-05-21

**Authors:** Maria Maj

**Affiliations:** Faculty of Foundry Engineering, AGH University of Krakow, Mickiewicza 30, 30-059 Krakow, Poland; mmaj@agh.edu.pl

**Keywords:** casting, strength of castings, residual stresses, operational stresses, thermal stresses, elasto-optics

## Abstract

This article presents some views on the subject of self- or residual stresses, trying to clarify some erroneously seemingly ingrained formulations in the introduction, which are widely used in castings and their classification (thermal, shrinkage, and phase stresses). For example, the location of their occurrence is often not specified, nor in which cross sections (volumes) they balance. In thin bars there are uniaxial stresses and in thin plates, stresses in two orthogonal directions are considered, while in castings, which are always three-dimensional objects, stresses in all planes should be considered. Meanwhile, to make matters worse, the complexity of calculations and possible experiments is rapidly increasing from the 1-axis to the 3-axis condition. A detailed analysis is made of how tensile and compressive stresses are calculated as a function of casting wall thickness, taking into account heat flow between walls of different thicknesses. The article presents selected methods of stress and strain testing, with particular emphasis on elasto-optical testing.

## 1. Introduction

The issue of residual stresses is extremely complex and at the same time, it is analyzed by many researchers in various aspects. The study of residual stresses has long been an important field of research in science and engineering because uncontrolled residual stresses have a harmful effect on the operation of elements. Numerous research works have been devoted to the quantification of residual stress states for the purposes of designing engineering components and predicting their service life and failure in service. Two main approaches can be distinguished, namely the interpretation of experimental measurements and process modeling. Most manufacturing processes induce residual stresses on the surface of the manufactured component. These residual stresses can be beneficial or, in some cases, detrimental to the performance of the component. Accurate determination of residual stresses plays a key role in understanding the complex interactions between microstructure, mechanical condition, failure mode(s), and structural integrity. Moreover, the concept of residual stress management contributes to industrial applications, aiming to improve service performance and product life cycle. Therefore, the industry requires fast, efficient, and modern methods for identifying and controlling the state of residual stresses [1,2,3,4,5,6,7,8,9]. 

The casting technique allows for giving practically any shape to designed products. In the process of strengthening, complex shapes pose significant difficulties, particularly in terms of analytical calculations, labor-intensive preparatory work for computer simulations, and possible model testing. This mainly concerns temperature fields and stress, deformation, and displacement fields, based on which the correctness of the design can be assessed.

Strengthening castings using modern specialized 3D computer programs and conducting strength evaluations of finished castings on load simulators can eliminate real threats resulting from design errors. Such procedures are of crucial importance in cyclically loaded components with variable stresses. In this type of problem, residual and operational stresses play a fundamental role, including cumulative thermal stresses, which, due to their complexity, are examined separately.

- Several misleading views have become widespread regarding stresses in castings, including the following. If the casting is significantly distorted, it is said to have large stresses. Indeed, they were the direct cause of the deformation but if present in a deformed and distorted casting, stresses are minimal. Rigid castings, such as wheels, can exhibit extreme stresses while maintaining their ideal shape;
- The magnitude of stresses changes over time (natural stress relief), so knowledge of the seasoning time, i.e., the history of the casting’s formation, is necessary;
- Testing the tendency of gray iron to form stresses is a technological test related to a specific casting, such as a grid with various cross-sectional rods, and has little to do with the examined casting of a different shape. The test helps evaluate the influence of chemical composition or other factors on the tendency to form stresses;
- For a designer, the most important factor is the distribution of residual and operational stresses due to the dangerous summation of stresses with the same signs (possibility of reaching the yield limit or even the ultimate strength);
- When considering stress issues, the location of their occurrence and the sections (volumes) in which they are balanced are often not specified. Uniaxial stresses occur in thin rods, biaxial stresses are considered in thin plates, whereas in castings, which are always three-dimensional objects, stresses should be considered in all planes. Moreover, to make matters worse, the level of complexity of calculations and potential experiments rapidly increases from a uniaxial state to a triaxial state.

The basis for stress evaluation in casting is its shape, size, manufacturing history, type of load during operation, and others. All of this affects the state of residual stresses and subsequent operational features. Both the impact of the casting’s shape as a whole and the arrangement of its individual elements are significant. Compact shapes such as blocks and spheres, as well as castings with cores, cool down slowly, which is why they are partially subjected to self-annealing. Plates, beams, and tubes cool down faster at the edges compared to the center. Conversely, according to the laws of thermodynamics, heat transfer always occurs through the surface, which, during cooling, has a lower temperature than the subsurface layers.

## 2. Evaluation of Stress Distribution in a Casting

Every raw casting, cooled to room temperature, is characterized by a specific state of residual stress. This state is the final result of temporary stress systems forming in the casting during solidification in the casting mold and, after removing it from the mold, until the temperature is equalized throughout the casting.

The main causes of residual stress formation in castings are the following:

- Uneven cooling, known as thermal restraint shrinkage—thermal stresses;
- Mechanical restraint shrinkage (by risers, cores, or rough surface)—shrinkage stresses;
- Structural non-uniformity, phase transformations, gas precipitation, bubbles, etc.—phase stresses [10,11,12,13,14].

Of course, these mentioned causes often occur together.

### 2.1. Thermal Stresses

Just as mentioned earlier, thermal stresses occur due to the uneven temperature distribution in different parts of the casting (thermal restraint shrinkage).

For example, in a simple rod fixed at both ends, when the temperature increases by Δ*T*, compressive stresses will occur according to the following formula:

*σ* = *α* Δ*TE* (1)

where

*α*—coefficient of thermal expansion;
Δ*T*—temperature increase;
*E*—longitudinal elastic modulus [MPa].

In a flat plate, with all edges clamped, when uniformly heated by Δ*T*, compressive stresses will be generated, as follows:

*σ* = Δ*T*
*α*
*E* (1 − *μ*) (2)

where *µ*—Poisson’s ratio.

If a straight rod is heated in such a way that the temperature on one surface is higher than the temperature on the other surface (assuming Δ*T* changes linearly), the rod will bend into an arc with a radius of curvature (3), as follows:

*ρ* = *h*/*α*Δ*T* (3)

where *h*—the height of the rod.

Clamping the ends of this rod will result in compressive stresses on the hotter side and tensile stresses on the colder side, as follows in Equation (4):

*σ* = *±*Δ*T*
*α*
*E* (4)

Similarly, a flat plate with unclamped edges, heated on one side by Δ*T*, will bend into a spherical surface with a radius of curvature (5), as follows:

*ρ* = *g*/*α*Δ*T* (5)

where *g*—thickness of the plate.

As before, clamping the ends of the plate will cause compressive and tensile stresses on the hotter and colder sides, respectively, according to Equation (6), as follows:

*σ* = *±*Δ*TαE*/2 (1 − *μ*) (6)

Assuming a hypothetical situation of two connected straight rods, made of the same material but with different thicknesses (forming one casting, so they must have the same length at each temperature), and assuming they cannot buckle, then in the initial state when the system transitions from a plastic state to an elastic state, the system will be free of stresses [15]. At this initial moment, the temperature difference between the thicker (slower cooling, therefore hotter) rod and the thinner rod is Δ*T*. After both rods cool down to the ambient temperature, stresses will be generated in them. The thicker rod will experience tensile stresses (7), as follows:

*σ*_2_ = +*F*_1_/(*F*_1_ + *F*_2_) *Eα*Δ*T* (7)

and in the faster cooling (thinner) compressive stress (8), as follows:

*σ*_1_ = −*F*_2_/(*F*_1_ + *F*_2_) *Eα*Δ*T* (8)

where *F*_1_—a cross-section of the thinner faster cooling rod and *F*_2_—a cross-section of the thicker slower cooling rod.

To determine the actual stress in thicker and thinner parts of the casting, it is necessary to take into account some phenomena such as gradual heat flow from thicker to thinner parts of the casting, partial occurrence of plastic deformation, unequal cooling rates of individual parts of the casting, temperature increase to a value at which the material transitions from the range of plastic deformations to elastic deformations, etc. [10].

The influence of these phenomena has been taken into account in Formulas (9)–(11), determining the stresses *σ*_1_ and *σ*_2_ in cast iron and steel castings [10].

*σ*_2_ = +*F*_1_/(*F*_1_ + *F*_2_) *M* (9)

*σ*_1_ = −*F*_2_/(*F*_1_ + *F*_2_) *M* (10)

where

(11) M=620αEKS1−620TpR2R1−1

where *σ*_1_ and *σ*_2_ are stresses in the thicker and thinner part of the casting (in the thicker and thinner rod), respectively. 

*α*—coefficient of linear expansion of the alloy in the elastic range (below 620 °C);

*K*—coefficient taking into account the conditions of heat flow between the thicker and thinner parts of the casting, whose value is between 1.0 and 2.0; the greater the thermal conductivity of the alloy and the larger the contact area of the two parts of the casting, the greater the coefficient;

*S*—coefficient taking into account the partial occurrence of plastic deformation, between 1.9 and 2.5, the larger the stress, the greater the coefficient;

*E*—modulus of the elasticity of cast iron;

*R*_2_ and *R*_1_—“effective” thicknesses of the thicker and thinner parts of the casting (thicker and thinner rod);

A 620 °C temperature at which iron alloys transition from the range of plastic deformations to the range of elastic deformations;

*T_p_*—pouring temperature (°C) [10].

The complexity of stress problems resulting from the variability of the coefficient of thermal expansion α and the modulus of longitudinal elasticity E with temperature complicates the issue of stress in castings. It can be concluded that the use of Hooke’s law in this case has limited application. Determining the relationship *E* = *f* (*T*) and α = *f* (*T*) for the entire temperature range from solidus temperature to ambient temperature is practically unattainable, which means that many problems related to optimal casting design still await a solution. The modulus of elasticity of cast iron decreases linearly with increasing stress. The correctness of the linear relationship between the modulus of elasticity and tensile stress was verified by A. Karamara [16,17] for gray and spheroidal cast iron. In the case of steel, no decrease in the modulus of elasticity with increasing stress is observed and its value remains practically constant. On the other hand, a comparison of the modulus of elasticity for pig iron and cast iron shows that there are no significant differences between them and only the fact that the modulus values for these species are very small deserves attention, which is undoubtedly related to the shape and size of graphite precipitates, which reach the largest sizes in the discussed cases. The value of the modulus decreases as the temperature increases, so a correction of deformation is necessary.

To summarize

- The value of thermal stresses does not depend on the length of the casting;
- The stresses in thicker and thinner walls of the casting are inversely proportional to the cross-sectional areas;
- The stresses are proportional to the modulus of elasticity of the material, so in steel castings, thermal stresses will be greater than in cast iron castings;
- Castings made of alloys with a high coefficient of expansion α should be expected to have high values of thermal stresses;
- Castings made of alloys with low thermal conductivity have large temperature differences, Δ*T*, in thick and thin parts of the casting and hence large values of thermal stresses.

Structural elements of castings such as thin protruding parts do not carry stresses, while their connections with the body, due to the clear change in cross-sections, create a structural notch, contributing to the accumulation of stresses. Holes made in a bent body away from the neutral axis are an additional negative factor, while when located on the neutral axis, they do not pose a threat.

### 2.2. Shrinkage Stresses

Due to the fact that shrinkage stresses are caused by mechanical restraint of shrinkage, they can only be tensile or compressive, both in thin and thicker sections, regardless of the cooling rate. Since shrinkage stresses occur in the temperature range corresponding to elastic deformation; if the cause of their formation is removed (e.g., by breaking the casting out of the mold, removing the core, etc.), these stresses should disappear but, in cases where plastic deformations occur regardless, they will remain in the casting. The restraint of shrinkage by the mold, core, or non-uniform temperature causes a difference in the cooling curves, resulting in deformations. In this case, the formula for stress must take into account the contribution of plastic stress (12), as follows:

*σ* = *ε*
*E* (1 − *δ*) (12)

where *δ*—contribution of plastic deformation.

In thin sections, tensile stresses occur, while compressive stresses occur in thick sections.

### 2.3. Phase Stresses

Phase stresses are caused by differences in the specific volume of individual structural components of the alloy, resulting from phase transformations (allotropic transformations, graphite formation in cast iron) in the elastic range. As the graphite formation increases with increasing thickness of the casting wall, the volume increment is greater in thicker parts than in thinner parts. Therefore, compressive stresses develop in thicker sections of the casting and tensile stresses develop in thinner sections, opposite in sign to thermal stresses. The summation of thermal stresses, caused by different values of cooling rates of the middle and outer layers and structural changes, results in the development of tensile stresses on the outer surface and compressive stresses inside the casting [12].

The sum of thermal, phase, and shrinkage stresses is referred to as residual stresses. Table 1 presents the types of stresses that develop in the casting depending on their location [10,12].

Residual stresses are also classified according to their location in which they balance each other.

- First kind, balancing throughout the casting, i.e., as much compressed volume as tensile volume;
- Second kind, balancing in small local areas, at a microscale of the elementary lattice, e.g., within grains, precipitates, inclusions, and gas bubbles;
- Third kind, considered within a deformed crystal lattice, with dislocations.

## 3. Methodology of Stress and Strain Analysis Applied to Castings

In general, to determine residual stresses, the strain caused by these stresses must be measured. For this purpose, complete or partial trepanation by division into elements or local stress relief by ring milling or small hole drilling must be performed. If the casting is equipped with design ribs, such as tubes, covers, and other thin-walled structures, stress relief is achieved by chamfering [10]. Strains are measured using previously attached resistance strain gauges and the measurement method is point-like. 

Experimental stress analysis carried out exclusively using resistance strain gauges, despite its advantages and availability, has a fundamental limitation: the measurement can only apply to a very small surface, equal to the surface of the strain gauge. In practical industrial applications, such a measurement is considered punctual. Therefore, this precise strain measurement is used in the second stage of research, after other methods are used to locate the primary directions of strain and stress concentrations. Surface methods include brittle coating methods, elastic optical coating techniques, holographic methods, moly strips, etc.

### 3.1. Brittle Coatings

The brittle coatings method involves applying a thin layer (0.025–0.1 mm) of specially prepared paint to a portion of the surface under investigation, which adheres tightly to the surface and deforms under load or unloading in the same way as the surface, resulting in characteristic cracks. Once fully dried, the paint adheres strongly to the surface and exhibits high brittleness, leading to the formation of cracks in the tensile stress zone. The cracks form perpendicular to the largest elongations. The order of their formation matches the local increase in local elongations. The distribution of compressive stresses is studied by applying the coating to the stressed surface and the cracks in the coating are obtained during unloading. 

There are many recipes and application technologies for brittle coatings. Several tests have also been developed to determine the strain sensitivity ε_0_ of the coating under uniaxial stress conditions (bending beam and tensile rod). For many recipes, the sensitivity of the coating ranges from 0.01 to 0.10%. The quality and accuracy of the measurement depend on several factors, including surface preparation procedures, coating application technique, drying, temperature and humidity, deformation rate, residual stresses, and others.

As a result of loading or unloading (in the case of residual stress analysis), the fine cracks of the brittle coating form a grid pattern, which represents the trajectories of one of the principal stresses. 

The direction of the cracks shown is perpendicular to the maximum relative deformation. The force direction that causes the cracks in this figure is vertical. The shape of the fractured area does not change, despite the increasing load, but it becomes larger and its boundaries are marked with load values from 16 to 40 kN. The lines marked in this way are called isoentat lines. Therefore, isoentates indicate the geometric location of points where the largest elongations have the same value. The appearance of cracks on the edge of the unstrained element, where one of the stresses is zero, also indicates the sensitivity of the brittle coating ε_0_. The strain sensitivity of brittle coatings is estimated at ε_0_ = (3–30) 10^−4^ [18].

**Figure 1:**
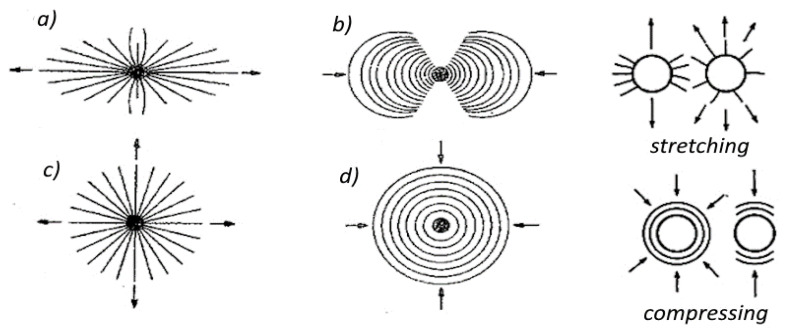
Directions of cracks in the vicinity of a notch in the form of a small drilled hole on the surface where there is (**a**) uniaxial tension, (**b**) uniaxial compression, (**c**) biaxial tension, and (**d**) biaxial compression. On the right are images of cracks caused by relaxation near the hole in the tensile and compressive regions (own research).

Images of brittle cracks formed near a hole under different loading conditions are shown in Figure 1.

The brittle coating method belongs to auxiliary qualitative methods. The measurement accuracy is not very high, for example, at stresses of the order of 100 to 200 MPa, errors can reach up to 25%. The use of factory aerosol lacquers and appropriate test procedures allows for a significant reduction in this error. Tens-lack coatings meet these requirements, as they are not only highly sensitive (5 × 10^−4^) but are also easy to apply in multiple layers, up to a thickness of 0.025 to 0.1 mm, and have distinct crack lines.

### 3.2. Method of the Elasto-Optical Layer

A number of examples characterizing stress and strain fields by elasto-optical methods (isochromes and isoclines images) with photostress and photoelast have been reviewed in the literature. Some of them are briefly described below. The purpose of the paper [19] was to analyze the stress fields generated when loads were applied to a notched specimen coated with an elasto-optical coating. The analysis was performed by the PhotoStress method using a reflectance polariscope and a digital video camera. On the notched tensile specimen, the differences in principal stresses at a specific point were determined at loads of 3 kN, 4.5 kN, 6 kN, 9 kN, and 12 kN. In addition, the value of the principal stress at the edge of the specimen at a load of 12 kN was determined and then compared with the numerical solution in SolidWorks. The difference between these values is 3.6%, which means that the experimental measurement was relatively precise. The works [20,21,22,23,24] emphasize that the use of an elasto-optical coating of the surface layer allows quantitative and qualitative analysis of the direction and magnitude of principal stresses and strains as a response to pressure loads on various structural components. Due to the versatility of this method, it can be used in many ways in various aspects of engineering projects, analysis of solutions to architectural structures, development of prosthetic implants, development of structural components of aircraft, machinery, etc. On the other hand, item [22] is devoted to the study of the stress states of a rotating body, which are created by the action of centrifugal force. In order to determine the distribution of stresses, the PhotoStress method was used, as well as a measuring device: the LF/Z-2 polariscope. The test specimen was subjected to loads of different magnitudes, which were recorded with the help of a photographic device. Another article [25] presents the selection and application of an elasto-optical coating on the surface of a selected 1-L irregularly shaped pressure vessel to determine the principal stresses and strains. It is worth noting that the role of prototypes, especially functional prototypes, has significantly increased [26], thanks to which it is possible to test selected components under operating conditions. In more complex cases, it is even worth conducting finite element analysis. Many works have compared the method of stress and strain analysis based on the image of isochromes and isoclines on the elasto-optical coating pasted on the real structure. Choosing the right research method for use in castings is half the battle, especially if one considers changes in the state of their surfaces caused by residual stresses. This issue concerns an object in which a series of shrinkage phenomena, changes in physico-mechanical properties during the cooling process, machining, etc., naturally occurred. They led to certain changes in shape and dimensions, indicating the presence of significant internal forces that are usually in balance. Disturbance of this system through the action of external forces, such as straightening the casting or its assembly, can even lead to cracking as a result of the sum of tensile stresses. Therefore, identifying the distribution of internal forces causing stresses becomes very important. However, obstacles such as the usually complex shape of the casting, its rigidity or structural flexibility, and the lack of simple calculation formulas make it difficult. In this situation, one or even several strain gauge measurements at various points on the casting do not give a satisfactory answer to questions regarding deformation across the entire surface. Even the brittle coatings method cannot satisfy matters, due to accessibility and difficulties in preparing the surface for testing, not to mention the difficulties in interpreting the obtained results [27].

However, there is a solution to the presented situation. Most foundries have well-equipped model shops where epoxy resin is used as an adhesive and gap filler and transparent plates with a thickness of 2 to 5 mm are made, which are necessary for the elasto-optical layer method, and gluing them onto the surfaces of castings would not pose any extraordinary difficulties. Creating and applying semi-polymerized coatings on curved surfaces would also not be too difficult. The only thing left is to obtain appropriate polarizing filters and start the research by recording images in the memory of a digital camera. The advantages brought by color digital photography and the ability to record results even in extremely difficult lighting conditions, as well as the immediate verification of the obtained effects, make the application possibilities and development of the optically active coatings method in the foundry industry seem obvious.

Elasto-optics uses the phenomenon of induced birefringence, which means that transparent materials become optically anisotropic under stress and deformation, and the resulting images overlap with the directions of the principal stresses. The scientific basis of elasto-optics is described in a series of monographs [28,29,30,31,32,33,34].

The method of optically sensitive coatings has an advantage over the classical method based on transparent models, in that it belongs to the methods of direct examination of real objects. It is worth noting that in the case of casts, with regard to objects that are generally thick compared to steel structures made of sheets and profiles, the highest stresses occur on the surface and in subsurface layers, so in both cases, the use of surface methods is justified. The interpretation of research results then requires ready equations from the theory of elasticity, regarding the state of stresses and strains in a flat state. Reflective measurements are carried out in the range of linear dependencies between the orders of isochromes, relative strains, and stresses in the elasto-optical layer. The strains in the layer can reach up to 3%, which also allows for measurements of plastic strains.

It is also worth noting the significant influence of the thickness of the casting walls on its shape strength and the material reserves inherent in optimal strength shaping, which can be largely taken into account in the examination using elasto-optical coatings. The easiest test can be carried out on a horizontal casting surface, which should be cleaned before starting the research. Epoxy adhesive with reflective aluminum dust is poured onto the prepared surface and spread evenly, maintaining a consistent thickness. Taking time (the adhesive curing time is several tens of minutes), any trapped air bubbles should be removed and a clean elasto-optical plate should be placed on top, ensuring no air is trapped underneath. The adhesive is applied to the vertical edges of the plate and then the excess is removed. Any accidental contamination with unpolymerized adhesive can still be removed and the area cleaned with a solvent. The examination is conducted after the complete curing of the adhesive, which is influenced by the amount of hardener added and the ambient temperature. 

**Figure 2:**
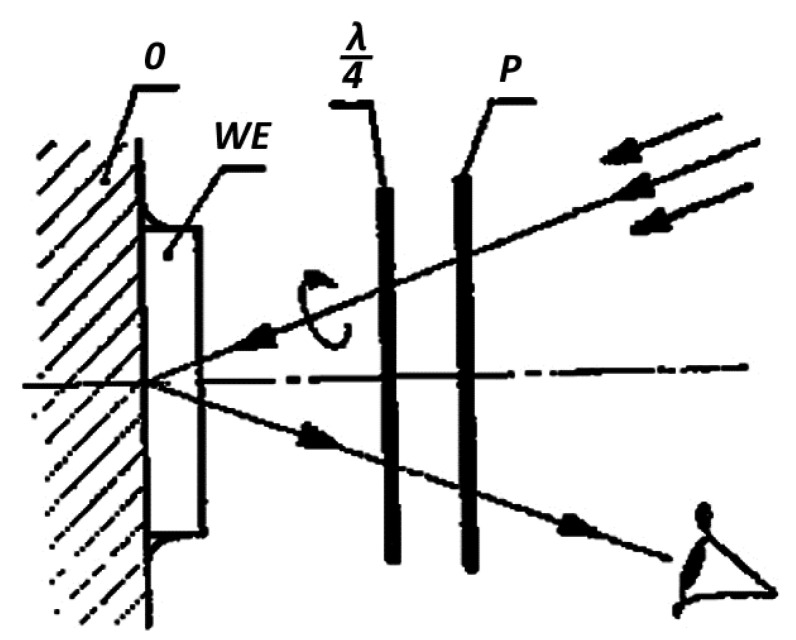
The simplest arrangement of a polariscope for light reflected from the object (own research).

Correctly attached coating observed in circularly polarized light should appear uniformly gray, without initial isochromes. Proper effects will occur if the object is subjected to external forces or internal stresses are released. In this case, white light passes through the polarization filter “P”, then the quarter-wave plate λ/4, where it is circularly polarized, penetrates twice through the elasto-optic plate “WE” because it reflects from the adhesive applied to the surface of the casting “O” (Figure 2). If, as a result of surface deformation in the plate, a birefringence effect in the form of colored fringes is produced, it is called an isochrome due to its constant color. They are geometric points at which the difference between the principal stresses has a constant value.

*σ*_1_ − *σ*_2_ = const (13)

or

*σ*_1_ − *σ*_2_ = m K (14)

where “m”—order of isochromes, indicated by consecutive digits 1, 2, and 3. “K”—model constant of stress dimension dependent on material “c”; coating thickness “t”; and wavelength λ. Isochromes are also geometric points of equal maximum shear stresses, i.e.,

*σ*_1_ − *σ*_2_ = 2 t_max_ (15)

Reversing the manual polariscope by 180 degrees eliminates the quarter-wave plate and then the polarized light is linear and dark lines appear against the background of colored isochromes, moving with the rotation of the polariscope. Those are lines along which the directions of stresses are the same and are called isoclines. The parameter of an isocline is the angle of inclination of one of the principal directions to the axis of the polariscope. Precise determination of this angle using a manual polariscope is troublesome, which is why another method of determining the direction of principal stresses is preferred, through drilling a hole and analyzing the resulting isochrome image. Such a procedure brings further benefits visible in the isochrome images shown in the subsequent drawings.

On the unloaded edges of the model and in places where uniaxial stress state occurs, one of the stresses is zero. In these locations, it is possible to directly determine the second principal stress, i.e., when *σ*_2_ = 0 then *σ*_1_ = mK.

Where t—layer thickness and fε—strain-optical coefficient of the layer material, which can be determined using a resistance strain gauge attached to the layer with isochromes.

**Figure 3:**
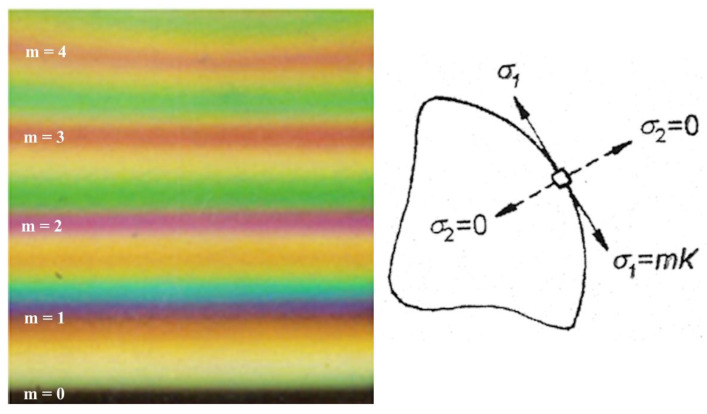
The sequence of colors corresponding to the total orders of isochromy starting from the dark m = 0, through yellow–red–blue–green m = 1, and further through yellow–red–green m = 2, etc., for higher orders (own research).

Figure 3 shows the order of occurrence of individual colors corresponding to the total orders of isochromy. The higher the order, the less intense the colors, especially above m = 3. For this reason, the parameters of the experiment are selected in such a way that the deformations do not exceed this value. The first order corresponds to the transition from red to blue, while higher orders are read at the red–green border.

**Figure 4:**
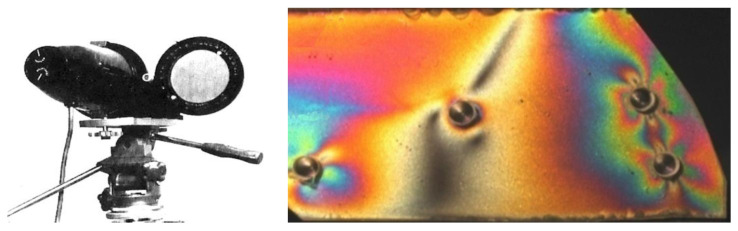
A professional reflective polariscope placed on a stand along with an example of an isochromic image obtained with its help (own research).

The polariscope shown in Figure 4 has two identical polarizing filters that are conjugated. The polarizer (on the left) serves as a reflector illuminating the surface under examination with polarized light, while the analyzer on the right allows for the examination of isochromic images along with fractional values and observations of isoclines and the registration of their parameters.

**Figure 5:**
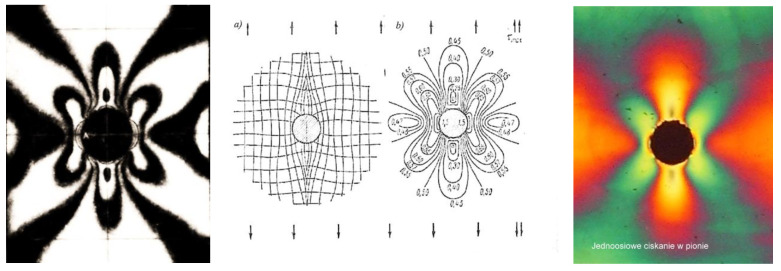
Isochromes in the vicinity of a uniaxially stretched flat bar are seen in sodium lamp light (on the **left**) and white light (on the **right**). The middle figures relate to the course of principal stress trajectories and maximum shear stress (own research).

**Figure 6:**
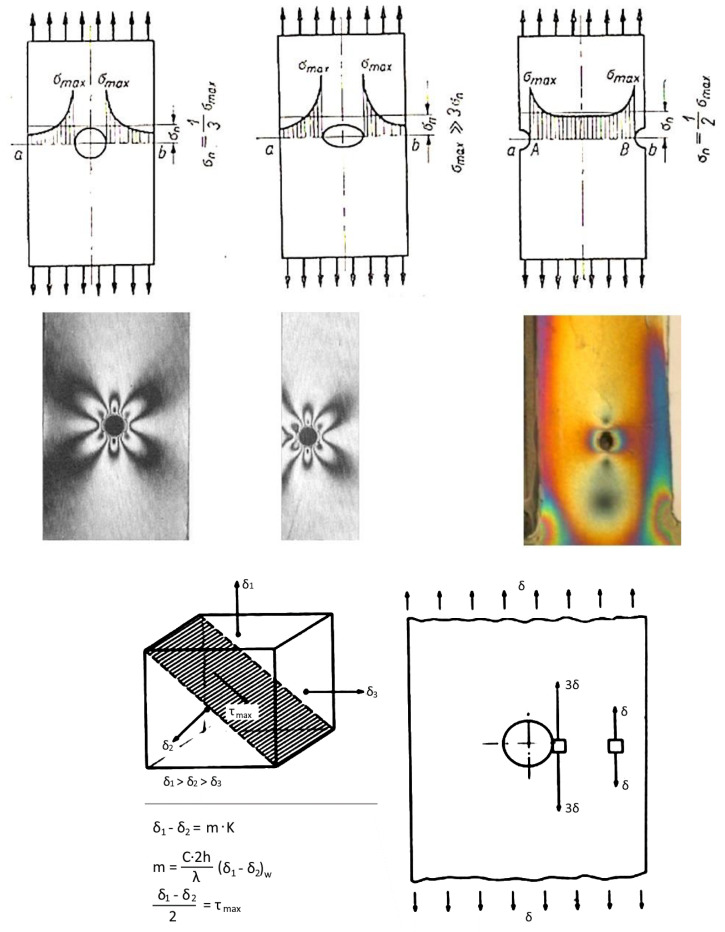
Examples of stress concentrations near a stretched flat bar with a hole (own research).

To create an isochromic image, a local deformation of the surface caused by loading or unloading must occur. In the case of studying internal stresses, local unloading with simultaneous accumulation of stresses occurs in the vicinity of a drilled small hole (2–5 mm) passing through the plate into the metal to a depth equal to the diameter of the drill. The effects of such action can be seen near the four holes shown in Figure 5. It presents the distribution of isochromes in a section of a curved beam resulting from bending, where tensile stresses occur on its outer side (stress accumulation at 2 holes) and compressive stresses on the inner side, while in the middle zone, a dark isochrome m = 0 appears. A more detailed interpretation of the images occurring near the hole during stress accumulation is shown in four drawings juxtaposed next to each other (Figure 6). The comparison of theory and practice (reality) is also presented in Figure 6, where stress accumulation is shown in a homogeneous stress field and near an unloaded edge.

As can be seen in Figure 5 and Figure 6, characteristic images appear near the hole, visible in polarized light, and based on them, a series of conclusions can be drawn about the state of stress in the examined area. Where the shape or dimensions of loaded elements change, the stress distribution changes; stresses accumulate and may be much greater than nominally calculated. The figure shows three notch failures and the elasto-optic images correlated with them (Figure 6). According to the theoretical solution (Kirsch), stress accumulation occurs at the edge of the hole, which, in the case of uniaxial stretching, is three times greater than the nominal stress. In addition, the shape of the isochrome near the hole does not change despite the increasing load. Therefore, the direction of principal stresses can be determined based on it. If an isochrome effect appears near the drilled hole, it indicates the existence of stress on the surface being tested. Otherwise, there is no stress [10,28].

Based on the orientation of the image, the direction of the greater of the principal stresses can be inferred. Ring-shaped isochromes around the drilled hole are formed when *σ*_1_ has the same value as *σ*_2_.

**Figure 7:**
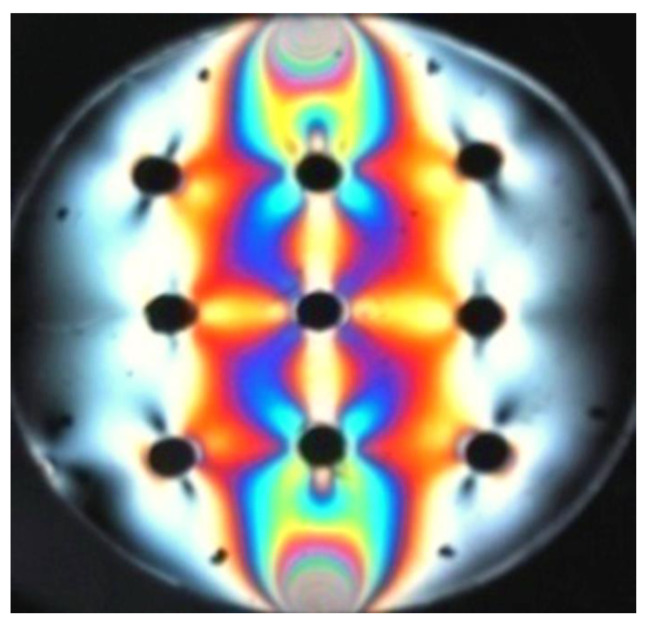
Compressed model with holes. The direction of greater stress, which is parallel to the contour of the disk, can be clearly seen in the external holes (own research).

Figure 7 shows a compressed disk with symmetrically distributed holes. The isochrome distribution allows for a series of conclusions regarding the stress distribution mentioned above, particularly, the absence of stress (dark field), direction of the principal stress (holes in the white field), maximum stresses (force application points), symmetry of deformations near the central hole, etc.

**Figure 8:**
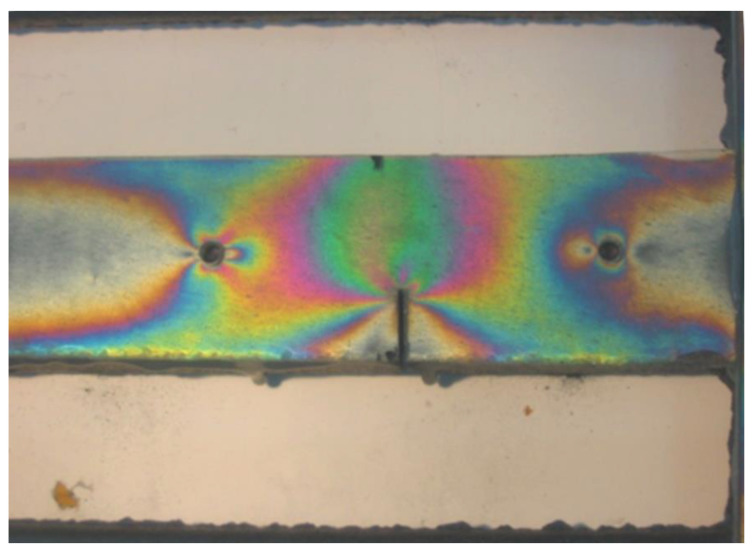
Deformations on a thick stress lattice bar during cutting, as well as effects near drilled holes (own research) [15,30].

**Figure 9:**
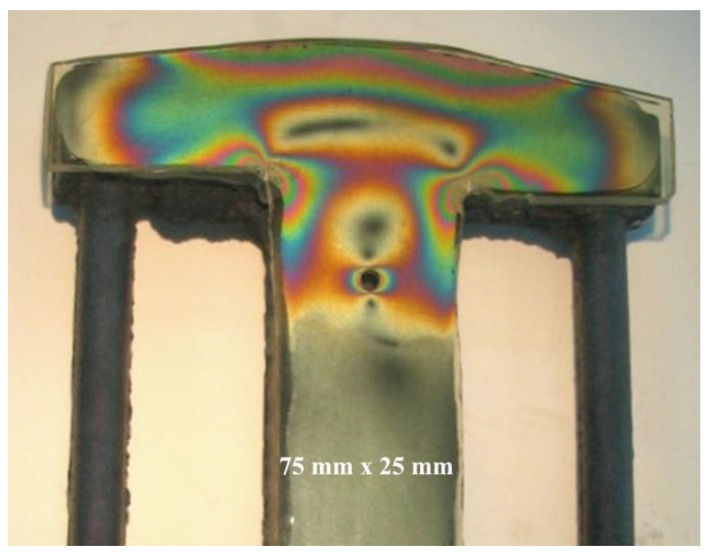
Isochrome image on a stress lattice casting after spontaneous cracking of a thick bar. The symmetry of the image is noteworthy, registering the deformation of the bent crossbar, the concentration of stresses in the corners, dark isochromes m = 0, and their accumulation near the drilled hole. Next to the lattice is the marked measurement point using the DMS method, which registered smaller deformations than those expected from the cracked section (own research) [15,30].

The occurrence of casting stresses in a so-called stress lattice is obvious. During the cutting of a thick bar with tensile stresses, spontaneous cracking occurs at some point and the magnitude of these residual stresses is measured by the size of this section. Covering the thick bar with an elasto-optic plate allows for tracking the distribution of isochromes during drilling and cutting, as shown in Figure 8. Drilling the holes before cutting indicated the stresses present in the lattice casting and indicated their direction, which was parallel to the edge of the bar. At the beginning of cutting the thick bar, there was a disturbance in the balance of forces in the lattice, which resulted in greater deformation in the weakened section than in the remaining sections. The resulting isochrome image indicates their concentration located at the front of the cut, while the corners of the cut bar are devoid of isochromes due to the release of stresses. Far from the cutting site, there is a homogeneous state of stresses, as evidenced by the lack of gradient in the color changes in the isochromes. The comparison of the two experiments shown in Figure 9 best demonstrates the advantages of the elasto-optic layer method in stress testing of castings.

**Figure 10:**
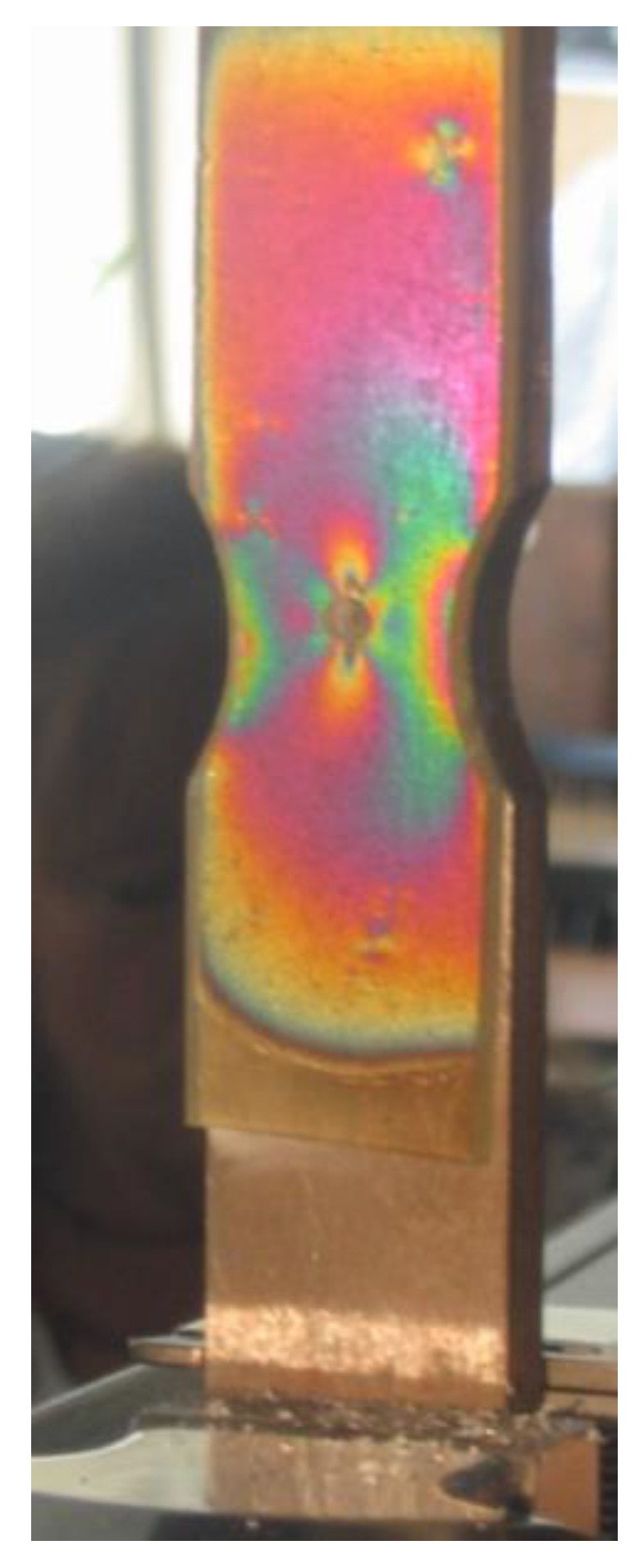
Isochromes on a stretched sample with a double carbide, visible in reflected light. A hole is drilled through the elasto-optic plate on the vertical axis. The obtained image near the hole indicates the direction of higher stress (own research) [15,30].

**Figure 11:**
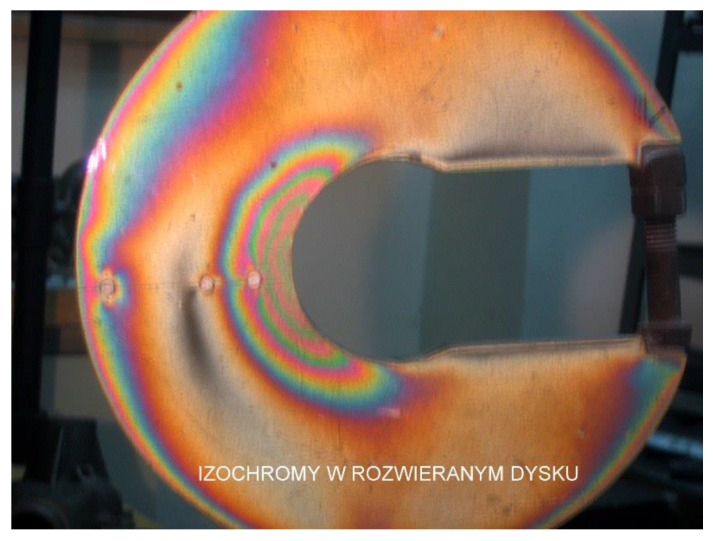
Isochromes on a stretched disc, visible in reflected light (own research) [15,30].

In stress and strain testing using elasto-optic methods, the type of transparent material with properly selected sensitivity is of great importance. A superficial observation of isochromes already indicates that the most distinct isochromes occur at low orders, up to m = 3. Additionally, the blue color is observed in the m = 1 range, which is absent at higher orders. This property facilitates the identification of stress growth directions. Another way to achieve the required level of isochrome order is the appropriate selection of plate thickness. However, with excessively thick plates, there is a significant reinforcement of the tested structure, which is essential for models made of organic glass. On the other hand, too-thin coatings require significant deformation to obtain the assumed optimal amount of isochromes, which is why the technique of applying coatings by spraying or painting has not found greater application. The most commonly used layer thicknesses (2 to 6 mm) are optically active enough and easy to produce technologically, in terms of their mechanical processing (cutting) and gluing (Figure 10 and Figure 11).

It is worth mentioning the advantage of the reflective method over the method of light passing through the model; as in the former, twice the sensitivity is achieved because the light passes through the birefringent layer twice.

The sensitivity of elastomeric materials, from which coatings or plates with a thickness of 2.0 to 5.0 mm for epoxy resins, polyester resins, and polycarbonates are cast, is comparable and their elasto-optic material constant is from 10 to 15 [kG/cm of the order of is]. Compared to soda-lime glass or polymethyl methacrylate, this sensitivity is about 20 times higher. Determining the order of isochromes is subject to certain rules. At the edges and sharp ends of the model, zero-order stresses can be expected unless it is loaded with forces in these areas. The numbers increase like contour lines, toward the accumulation near the carbide. Between them, there are half-order isochromes, while fractional orders can be obtained by compensation methods. Figure 3 shows the increasing orders of isochromes, which appear successively with increasing load of the plate with a hole.

Epoxy resin materials in the cured state are stiff with E = 30,000 [kG/cm^2^] and a Poisson’s ratio of ν = 0.3. The formulation of their preparation requires the use of various additives in the form of solvents, plasticizers, and modifiers, which affects the final properties; therefore, they require characterization.

During the preparation of elasto-optic materials, their polymerization, and mechanical processing, heat is released, which must be dissipated. An increase in temperature accelerates the reaction of curing the liquid composition and weak thermal conductivity hinders its homogenization in the entire mass, which is why the one-time amount of prepared resin is limited. However, the weak thermal conductivity of plastics is used in the study of thermal stresses on the housings of thermal machines on which they act.

## 4. Holographic Recording of Displacements

**Figure 12:**
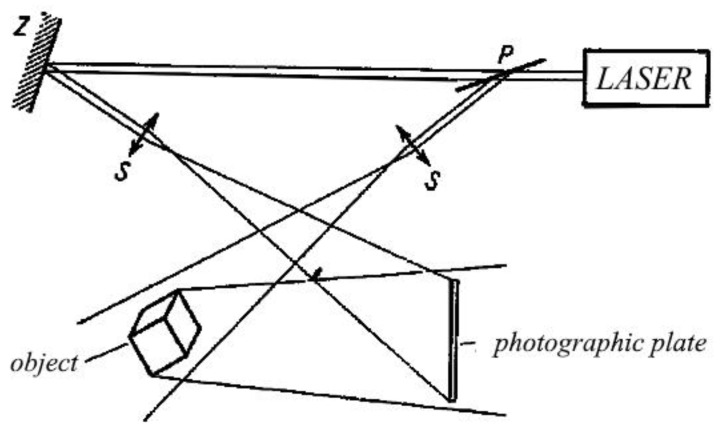
Diagram of the laser beam path (P–semi-transparent mirror and Z–lenses) (own research).

**Figure 13:**
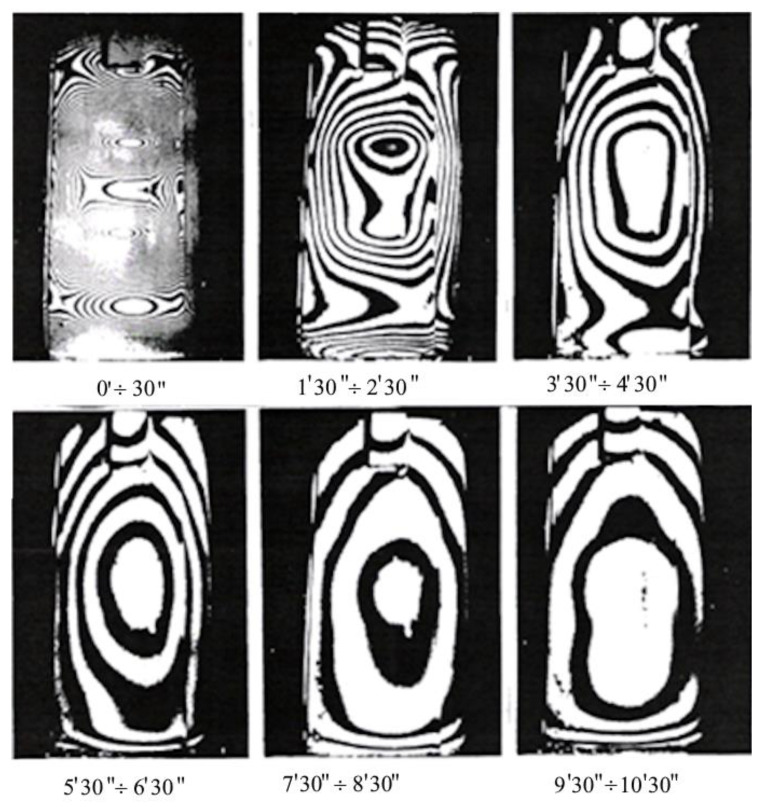
Holograms of the ladle model were obtained using the double exposure method at time intervals described below the photograph (own research).

**Figure 14:**
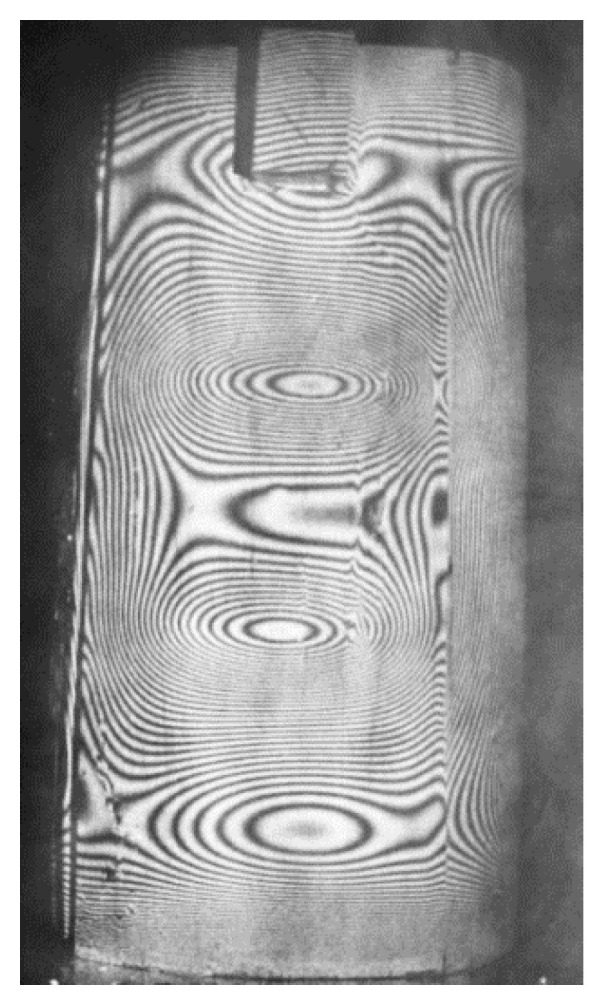
Interference fringes on the surface of the steel ladle model in the initial heating phase. Visible singular points are related to the construction and operational durability of the ladle (own research).

Displacements of the surfaces of examined castings can be caused by various factors, such as temperature changes, pressure, applied loads from external forces, and release of own stresses by local unloading (edge cutting, hole drilling, ring milling, etc.). These displacements, related to deformations and thus stresses, are generally small and difficult to measure using traditional methods. This statement applies to die molds, hot core boxes, or other metal forms, where complex thermal–mechanical loads may occur. In such cases, surface contactless examination methods are recommended, which, compared to point methods, provide rich material for the analysis of occurring phenomena, even only in terms of quality [31]. For studying the kinetics of surface displacements in specific time intervals, the holographic method of double exposure is best applied. A simple setup for creating and reconstructing holograms consists of several optical elements such as:

- A beam director and beam splitter for laser light into the object and reference beams;
- An illuminating mirror for the tested object. The light scattered by the tested surface, covered with magnesium oxide for better contrast, falls on a hologram, i.e., a photosensitive plate with very high resolution, where it interferes with the reference beam directed by the mirror onto the hologram. The diagram of the arrangement for creating a hologram is shown in Figure 12, while the next Figure 13 and Figure 14 show an example of the superposition of interference fringes in real-time, the thermally stabilized initial state, and the state induced by the thermal load of the plastic model of the runner within 30s from the beginning of heating.

Subsequent photographs show interference fringes formed as a result of exposure at later stages of the heating process while maintaining the same time interval between the first and second exposure (equal time intervals of 1 min). The most interesting is the first hologram representing the thermal shock, revealing extreme displacements and the influence of the weight of the lower part of the model. For better readability, Figure 14 shows an enlargement of the initial heating period image, which precisely revealed the fact of non-uniform displacements of the model surface during the thermal shock. This was achieved thanks to the high sensitivity of measurement equal to the wavelength of the helium-neon laser and the photographic plate with a resolution of several thousand lines per millimeter. The modern digital recording provides broader opportunities for adapting holography to engineering tasks.

Holographic interferometry methods are used in qualitative and quantitative studies of strains and stresses in various types of objects, such as structural elements, machine parts, various materials in any processing state, etc. Qualitative analysis of the images obtained using holographic interferometry allows for the identification and location of internal cracks and delaminations and heterogeneity of the tested material.

## 5. Other Methods of Stress Analysis in Castings

These include methods that utilize hardness measurement, related to the plastic deformations of the tested surface during indentation. The use of appropriate standards and charts allows for the assessment of stress levels. More accurate results are obtained using methods utilizing neutron beams or X-rays. Here, the measurement of atomic plane distances is performed using the phenomenon of diffraction and Bragg’s law for the reflected beams from the tested surface. The needs of the foundry industry are completely satisfied by the methods mentioned earlier, namely resistive strain gauge method and optical methods, such as reflective elasto-optics and holography.

## 6. Conclusions

The briefly presented experimental testing methods are primarily for qualitative analysis of deformations that respond to given loads. This is especially with regard to when it comes to elasto-optical (model) testing but also with respect to those using elasto-optical coatings applied to an existing casting or structure. It may be worth emphasizing the significant influence of the thickness of the casting walls on its shape strength and the material reserves inherent in optimal strength shaping, which can be largely taken into account, e.g., in testing the elasto-optical coverings. In the period immediately preceding the computer age, a method for rapid experimental prototyping was developed. With an elasto-optical coating glued onto an actual component, a model/actual structure under load can be observed. Based on the analysis of the elasto-optical images, the correctness of the design can be inferred and the optimization of the design is reduced to adding or removing material during the prototype improvement procedure. Nowadays, it is possible to cross-verify the methods of experimental shaping of casting with calculations based at least on the finite element method [FEM]. [34,35,36,37,38,39,40]

## 7. Future Directions

In the future, it is planned to make a series of prototype models of various cast components, verified experimentally with simultaneous finite element calculations. An example of a comparison between elasto-optical and computational (FEA) tests can be found in publication [41], which presents the results of elasto-optical tests on drilled specimens and compares them with those carried out using finite element analysis. Polycarbonate specimens were subjected to tension and on an elasto-optical polariscope; isochromic lines were observed. The same specimens made of Al7075 alloy (used for aerospace structures) were subjected to finite element analysis and the results of both tests were successfully compared.

## Figures and Tables

**Table 1:** Types of stresses that develop in thick and thin sections of cooling castings.

Characteristic of Casting Part	Thermal Stresses	Shrinkage Stresses		Phase Stresses	
		In the Form	After Being Taken out of the Form	*γ* *→* *α Transformation*	*Pre-Eutectoid Graphite Formation + + − or* 0 *±*
*In thick sections and internal layers of the casting*	+	+	**−** or **0**	±	**−**
*In thin sections and outer layers of the casting*	**−**	+	**+** or **0**	±	+

Note: “+” indicates tensile stresses and “−” indicates compressive.

## Data Availability

Data are contained within the article.
